# West Nile Virus and Other Arboviral Diseases — United States, 2013

**Published:** 2014-06-20

**Authors:** Nicole P. Lindsey, Jennifer A. Lehman, J. Erin Staples, Marc Fischer

**Affiliations:** 1 Division of Vector-Borne Diseases, National Center for Emerging and Zoonotic Infectious Diseases, CDC

Arthropod-borne viruses (arboviruses) are transmitted to humans primarily through the bites of infected mosquitoes and ticks. West Nile virus (WNV) is the leading cause of domestically acquired arboviral disease in the United States (1). However, several other arboviruses also cause sporadic cases and seasonal outbreaks of neuroinvasive disease (i.e., meningitis, encephalitis, and acute flaccid paralysis) (1). This report summarizes surveillance data reported to CDC in 2013 for WNV and other nationally notifiable arboviruses, excluding dengue. Forty-seven states and the District of Columbia reported 2,469 cases of WNV disease. Of these, 1,267 (51%) were classified as WNV neuroinvasive disease, for a national incidence of 0.40 per 100,000 population. After WNV, the next most commonly reported cause of arboviral disease was La Crosse virus (LACV) (85 cases), followed by Jamestown Canyon virus (JCV) (22), Powassan virus (POWV) (15), and eastern equine encephalitis virus (EEEV) (eight). WNV and other arboviruses continue to cause serious illness in substantial numbers of persons annually. Maintaining surveillance remains important to help direct and promote prevention activities.

In the United States, most arboviruses are maintained in transmission cycles between arthropods and vertebrate hosts (typically birds or small mammals). Humans usually become infected when bitten by infected mosquitoes or ticks. Person-to-person transmission occurs rarely through blood transfusion and organ transplantation. Most human arboviral infections are asymptomatic. Symptomatic infections most often manifest as a systemic febrile illness and, less commonly, as neuroinvasive disease. Most endemic arboviral diseases are nationally notifiable and are reported to CDC through the ArboNET surveillance system (2,3). In addition to collecting data on human disease cases, ArboNET collects data on viremic blood donors, veterinary disease cases, and infections in mosquitoes, dead birds, and sentinel animals. Using standard definitions, human cases with laboratory evidence of recent arboviral infection are classified as neuroinvasive disease or nonneuroinvasive disease (2). Because of the substantial associated morbidity, detection and reporting of neuroinvasive disease cases is assumed to be more consistent and complete than for nonneuroinvasive disease cases. Therefore, incidence rates were calculated for neuroinvasive disease cases using U.S. Census Bureau 2013 mid-year population estimates.

In 2013, CDC received reports of 2,605 cases of nationally notifiable arboviral diseases, including those caused by WNV (2,469 cases), LACV (85), JCV (22), POWV (15), EEEV (eight), unspecified California serogroup virus (five), and St. Louis encephalitis virus (SLEV) (one). Cases were reported from 830 (26%) of the 3,141 U.S. counties; no cases were reported from Alaska or Hawaii. Of the 2,605 arboviral disease cases, 1,383 (53%) were reported as neuroinvasive disease, for a national incidence of 0.44 per 100,000 population.

A total of 2,469 WNV disease cases, including 1,267 (51%) neuroinvasive cases, were reported from 725 counties in 47 states and the District of Columbia (Table 1). WNV disease cases peaked in early September; 90% of cases had illness onset during July–September. The median age of patients was 55 years (interquartile range [IQR] = 41–67 years); 1,425 (58%) were male. Overall, 1,494 (61%) patients were hospitalized, and 119 (5%) died. The median age of patients who died was 78 years (IQR = 67–83 years).

Of the 1,267 WNV neuroinvasive disease patients, 669 (53%) had encephalitis, 486 (38%) had meningitis, and 112 (9%) had acute flaccid paralysis. Among the 112 patients with acute flaccid paralysis, 85 (76%) also had encephalitis or meningitis. The national incidence of neuroinvasive WNV disease was 0.40 per 100,000 population (Table 2). States with the highest incidence rates included North Dakota (8.9 per 100,000), South Dakota (6.8), Nebraska (2.9), and Wyoming (2.8) (Figure). Six states reported approximately half (51%) of the WNV neuroinvasive disease cases: California (237 cases), Texas (113), Colorado (90), Illinois (86), North Dakota (64), and Oklahoma (60). Neuroinvasive WNV disease incidence increased with age, with the highest incidence among persons aged ≥70 years. Among patients with neuroinvasive disease, 111 (9%) died.

The 85 LACV disease cases were reported from 59 counties in 12 states; 77 (91%) were neuroinvasive (Table 1). Dates of illness onset for LACV disease cases ranged from June through October; 71 (84%) had onset during July–September. Forty-nine (58%) patients were male. The median age of patients was 7 years (IQR = 4–11 years); 76 (89%) were aged <18 years. LACV neuroinvasive disease incidence was highest in West Virginia (0.54 per 100,000), Tennessee (0.35), North Carolina (0.13), and Ohio (0.12) (Table 2). Those four states reported 60 (78%) LACV neuroinvasive disease cases. A total of 80 (94%) patients were hospitalized; two (2%) died.

Twenty-two JCV disease cases were reported from 20 counties in 10 states; 15 (68%) were neuroinvasive (Table 1). Eight states (Georgia, Idaho, Massachusetts, Minnesota, New Hampshire, Oregon, Pennsylvania, and Rhode Island) reported their first JCV disease cases. Dates of illness onset ranged from January through November, with 14 (64%) of the 22 cases occurring during July–September. The median age of patients was 46 years (IQR = 32–57 years); 17 (77%) were male. Twelve (55%) patients were hospitalized; none died. In addition to the LACV and JCV cases, five cases of California serogroup virus disease were reported for which the specific infecting virus was unknown.

Fifteen POWV disease cases were reported from 13 counties in seven states; 12 (80%) were neuroinvasive (Table 1). Three states (Massachusetts, New Hampshire, and New Jersey) reported their first POWV disease cases. Dates of illness onset ranged from May through November; five (33%) had onset during April–June, and six (40%) had onset during July–September. The median age of patients was 69 years (IQR = 45–75 years); 11 (73%) were male. Thirteen (87%) patients were hospitalized; two (13%) died.

Eight EEEV disease cases were reported from six states, including the first cases ever reported from Arkansas and Connecticut (Table 1). All eight were neuroinvasive. Dates of illness onset ranged from January through December. The median age of patients was 56 years (IQR = 33–74 years); six (75%) were male. All eight patients were hospitalized; four (50%) died. The median age of patients who died was 62 years (IQR = 33–86 years).

One SLEV neuroinvasive disease case was reported from Texas; the patient was hospitalized and survived.

## Discussion

In 2013, WNV was the most common cause of neuroinvasive arboviral disease in the United States. However, LACV was the most common cause of neuroinvasive arboviral disease among children. More JCV cases were reported in 2013 than in any previous year and included the first cases reported from eight states. This increase is likely related to the initiation of routine immunoglobulin M testing for JCV at CDC in 2013 and suggests that the incidence of JCV infection in prior years might have been underestimated. EEEV disease, although rare, remained the most severe arboviral disease, with four deaths among eight patients. More than 90% of arboviral disease cases occurred during April–September, emphasizing the importance of focusing public health interventions during this period.

Reported numbers of arboviral disease cases vary from year to year. Weather (e.g., temperature and precipitation), zoonotic host and vector abundance, and human behavior (e.g., repellent use, outdoor activities, and use of air conditioning or screens in the home) are all factors that can influence when and where outbreaks occur. This complex ecology makes it difficult to predict how many cases of disease might occur in the future and where they will occur. Increased numbers of reported cases and the identification of cases in new locations might reflect actual changes in incidence and epidemiology or increased disease awareness.

The incidence of WNV neuroinvasive disease declined substantially in 2013 (incidence of 0.40 per 100,000 population) compared with 2012 (0.92 per 100,000 population), when a large multistate outbreak occurred, with incidence nearing the levels observed in 2002 and 2003 (4). However, the incidence in 2013 was similar to that during 2004–2007 (median = 0.43; range = 0.39–0.50) and was higher than that during 2008–2011 (median = 0.18; range: 0.13–0.23) (3–5). WNV activity remained focalized in 2013, with more than half of the neuroinvasive disease cases being reported from just six states.

The findings in this report are subject to at least two limitations. First, ArboNET is a passive surveillance system that relies on clinicians to consider the diagnosis of an arboviral disease and obtain appropriate diagnostic tests, and on health-care providers and laboratories to report laboratory-confirmed cases to public health authorities. Second, testing and reporting are incomplete, leading to a substantial underestimate of the actual number of cases (6). For example, data from previous studies suggest there are 30–70 nonneuroinvasive disease cases for every reported case of WNV neuroinvasive disease (7–9). Extrapolating from the 1,267 WNV neuroinvasive disease cases reported, an estimated 38,000–88,500 nonneuroinvasive disease cases might have occurred in 2013. However, only 1,202 (1%–3%) were diagnosed and reported.

What is already known on this topic?

West Nile virus (WNV) is the leading cause of domestically acquired arboviral disease in the United States. However, several other arboviruses can cause sporadic cases and outbreaks of neuroinvasive disease, mainly in the summer.

What is added by this report?

In 2013, WNV was the most common cause of neuroinvasive arboviral disease in the United States (1,267 cases). However, La Crosse virus was the most common cause of neuroinvasive arboviral disease among children. More Jamestown Canyon virus disease cases (22) were reported in 2013 than in any previous year and included the first cases reported from eight states. Eastern equine encephalitis virus disease, although rare, remained the most severe arboviral disease, with a 50% case-fatality ratio.

What are the implications for public health practice?

WNV and other arboviruses continue to be a source of severe illness each year for substantial numbers of persons in the United States. Maintaining surveillance remains important to identify outbreaks and guide prevention efforts. Prevention efforts depend upon applying insecticides, reducing mosquito breeding grounds, use of repellents, and wearing protective clothing.

Arboviruses continue to cause substantial morbidity in the United States. However, cases occur sporadically, and the epidemiology varies by virus and geographic area. Surveillance is essential to identify outbreaks and guide prevention efforts aimed at reducing the incidence of these diseases. Health-care providers should consider arboviral infections in the differential diagnosis of cases of aseptic meningitis and encephalitis, obtain appropriate specimens for laboratory testing, and promptly report cases to public health authorities (2). Because human vaccines against domestic arboviruses are not available, prevention of arboviral disease depends on community and household efforts to reduce vector populations (e.g., applying insecticides and reducing mosquito breeding sites), personal protective measures to decrease exposure to mosquitoes and ticks (e.g., use of repellents and wearing protective clothing), and screening blood donors.

## Figures and Tables

**FIGURE:**
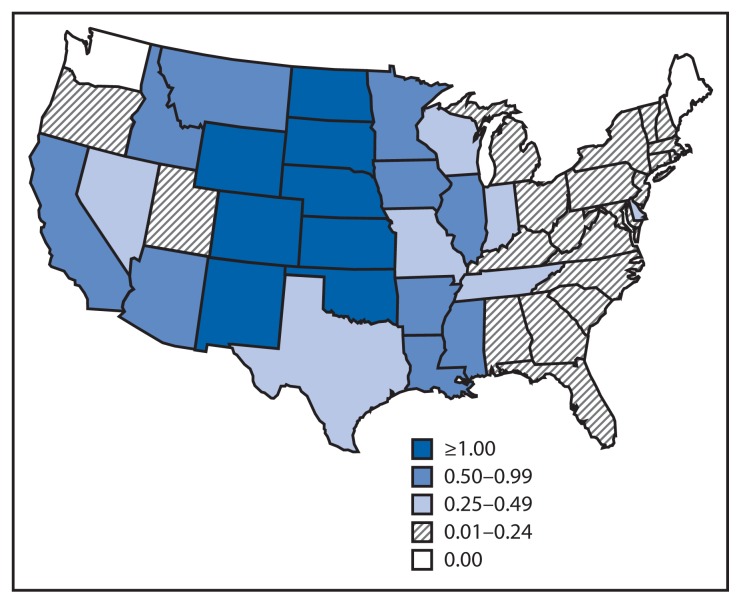
Incidence* of reported cases of West Nile virus neuroinvasive disease, by state — United States, 2013 * Per 100,000 population, based on July 1, 2013 U.S. Census population estimates.

**TABLE 1:** Number and percentage of reported cases of arboviral disease, by virus and selected characteristics — United States, 2013*

	Virus									
	
	West Nile (n = 2,469)		La Crosse (n = 85)		Jamestown Canyon (n = 22)		Powassan (n = 15)		Eastern equine encephalitis (n = 8)	
					
Characteristic	No.	(%)	No.	(%)	No.	(%)	No.	(%)	No.	(%)
**Age group (yrs)**										
<18	96	(4)	76	(89)	3	(14)	1	(7)	2	(25)
18–59	1,392	(56)	6	(7)	16	(73)	6	(40)	3	(38)
≥60	981	(40)	3	(4)	3	(14)	8	(53)	3	(38)
**Sex**										
Male	1,425	(58)	49	(58)	17	(77)	11	(73)	6	(75)
Female	1,044	(42)	36	(42)	5	(23)	4	(27)	2	(25)
**Period of illness onset**										
January–March	4	(<1)	0	(0)	1	(5)	0	(0)	2	(25)
April–June	49	(2)	9	(11)	5	(23)	5	(33)	1	(13)
July–September	2,223	(90)	71	(84)	14	(64)	6	(40)	2	(25)
October–December	193	(8)	5	(6)	2	(9)	4	(27)	3	(38)
**Clinical syndrome**										
Nonneuroinvasive	1,202	(49)	8	(9)	7	(32)	3	(20)	0	(0)
Neuroinvasive	1,267	(51)	77	(91)	15	(68)	12	(80)	8	(100)
Encephalitis	669	(27)	65	(76)	9	(41)	10	(67)	8	(100)
Meningitis	486	(20)	8	(9)	6	(27)	2	(13)	0	(0)
Acute flaccid paralysis†	112	(5)	4	(5)	0	(0)	0	(0)	0	(0)
**Outcome**										
Hospitalization	1,494	(61)	80	(94)	12	(55)	13	(87)	8	(100)
Death	119§	(5)	2	(2)	0	(0)	2	(13)	4	(50)

* Five unspecified California serogroup virus disease cases in addition to the La Crosse virus and Jamestown Canyon virus disease cases were reported.

† Of the 112 West Nile virus disease patients with acute flaccid paralysis, 85 (76%) also had encephalitis or meningitis. The four La Crosse virus disease patients with acute flaccid paralysis all also had encephalitis.

§ Of the 119 West Nile virus deaths, 111 (93%) occurred in patients with neuroinvasive disease and eight (7%) in patients with nonneuroinvasive disease.

**TABLE 2:** Number and rate* of reported cases of arboviral neuroinvasive disease, by virus, U.S. Census region, and state — United States, 2013

	Virus									
	
	West Nile		La Crosse		Jamestown Canyon		Powassan		Eastern equine encephalitis	
					
U.S. Census region/State	No.	Rate	No.	Rate	No.	Rate	No.	Rate	No.	Rate
**United States**	**1,267**	**0.40**	**77**	**0.02**	**15**	**<0.01**	**12**	**<0.01**	**8**	**<0.01**
**New England**	**11**	**0.08**	**—**	**—**	**3**	**0.02**	**3**	**0.02**	**2**	**0.01**
Connecticut	1	0.03	—	—	—	—	—	—	1	0.03
Maine	—	—	—	—	—	—	1	0.08	—	—
Massachusetts	7	0.10	—	—	1	0.01	1	0.01	1	0.01
New Hampshire	1	0.08	—	—	1	0.08	1	0.08	—	—
Rhode Island	1	0.10	—	—	1	0.10	—	—	—	—
Vermont	1	0.16	—	—	—	—	—	—	—	—
**Middle Atlantic**	**34**	**0.08**	**—**	**—**	**3**	**0.01**	**5**	**0.01**	**—**	**—**
New Jersey	10	0.11	—	—	—	—	1	0.01	—	—
New York	18	0.09	—	—	3	0.02	4	0.02	—	—
Pennsylvania	6	0.05	—	—	—	—	—	—	—	—
**East North Central**	**167**	**0.36**	**20**	**0.04**	**7**	**0.02**	**3**	**0.01**	**—**	**—**
Illinois	86	0.67	—	—	—	—	—	—	—	—
Indiana	19	0.29	1	0.02	—	—	—	—	—	—
Michigan	24	0.24	—	—	—	—	—	—	—	—
Ohio	21	0.18	14	0.12	—	—	—	—	—	—
Wisconsin	17	0.30	5	0.09	7	0.12	3	0.05	—	—
**West North Central**	**288**	**1.38**	**4**	**0.02**	**1**	**0.00**	**1**	**0.00**	**—**	**—**
Iowa	24	0.78	—	—	—	—	—	—	—	—
Kansas	34	1.17	—	—	—	—	—	—	—	—
Minnesota	31	0.57	4	0.07	1	0.02	1	0.02	—	—
Missouri	24	0.40	—	—	—	—	—	—	—	—
Nebraska	54	2.89	—	—	—	—	—	—	—	—
North Dakota	64	8.85	—	—	—	—	—	—	—	—
South Dakota	57	6.75	—	—	—	—	—	—	—	—
**South Atlantic**	**36**	**0.06**	**27**	**0.04**	**—**	**—**	**—**	**—**	**5**	**0.01**
Delaware	3	0.32	—	—	—	—	—	—	—	—
District of Columbia	—	—	—	—	—	—	—	—	—	—
Florida	5	0.03	—	—	—	—	—	—	3	0.02
Georgia	4	0.04	1	0.01	—	—	—	—	1	0.01
Maryland	11	0.19	—	—	—	—	—	—	—	—
North Carolina	3	0.03	13	0.13	—	—	—	—	1	0.01
South Carolina	3	0.06	1	0.02	—	—	—	—	—	—
Virginia	6	0.07	2	0.02	—	—	—	—	—	—
West Virginia	1	0.05	10	0.54	—	—	—	—	—	—
**East South Central**	**48**	**0.26**	**26**	**0.14**	**—**	**—**	**—**	**—**	**—**	**—**
Alabama	3	0.06	1	0.02	—	—	—	—	—	—
Kentucky	1	0.02	—	—	—	—	—	—	—	—
Mississippi	27	0.90	2	0.07	—	—	—	—	—	—
Tennessee	17	0.26	23	0.35	—	—	—	—	—	—
**West South Central**	**223**	**0.59**	**—**	**—**	**—**	**—**	**—**	**—**	**1**	**<0.01**
Arkansas	16	0.54	—	—	—	—	—	—	1	0.03
Louisiana	34	0.74	—	—	—	—	—	—	—	—
Oklahoma	60	1.56	—	—	—	—	—	—	—	—
Texas	113	0.43	—	—	—	—	—	—	—	—
**Mountain**	**216**	**0.94**	**—**	**—**	**—**	**—**	**—**	**—**	**—**	**—**
Arizona	50	0.75	—	—	—	—	—	—	—	—
Colorado	90	1.71	—	—	—	—	—	—	—	—
Idaho	14	0.87	—	—	—	—	—	—	—	—
Montana	10	1.00	—	—	—	—	—	—	—	—
Nevada	8	0.29	—	—	—	—	—	—	—	—
New Mexico	24	1.15	—	—	—	—	—	—	—	—
Utah	4	0.14	—	—	—	—	—	—	—	—
Wyoming	16	2.75	—	—	—	—	—	—	—	—
**Pacific**	**244**	**0.47**	**—**	**—**	**1**	**<0.01**	**—**	**—**	**—**	**—**
Alaska	—	—	—	—	—	—	—	—	—	—
California	237	0.62	—	—	—	—	—	—	—	—
Hawaii	—	—	—	—	—	—	—	—	—	—
Oregon	7	0.18	—	—	1	0.03	—	—	—	—
Washington	—	—	—	—	—	—	—	—	—	—

* Per 100,000 population, based on July 1, 2013 U.S. Census population estimates.
